# Data on tumor progression of *c-mos* deficiency in murine models of *Kras*^*G12D*^ lung and *Apc*^*Min*^ colorectal cancer

**DOI:** 10.1016/j.dib.2018.08.129

**Published:** 2018-08-31

**Authors:** Zhengxi Chen, Ju Qiao, Qirui Wang, Qian Xiao

**Affiliations:** a Department of Pharmacology, School of Medicine, Yale University, 10 Amistad St, New Haven, CT, USA; b Department of Mechanical and Industrial Engineering, Northeastern University, Boston, MA, USA; c Department of Molecular Biology, State Administration of Traditional Chinese Medicine, School of Traditional Chinese Medicine, Southern Medical University, Guangzhou, China; d Department of Orthodontics, Shanghai Ninth People׳s Hospital, School of Stomatology, Shanghai key Laboratory of Stomatology, Shanghai Jiao Tong University, Shanghai, China

## Abstract

The *c-mos* proto-oncogene was one of the first proto-oncogenes to be cloned. Apart from its role in meiosis, many efforts have been made to illuminate the mechanisms by which *c-mos* might acts as an oncogene. Increased Mos expression was found in most human tumor tissues. However, a detailed role of *c-mos* in tumor progression remains unknown.

In this study, we analyzed online databases to find out the correlation between Mos expression and poor survival rates in human cancer patients. Then, we crossed *c-mos* knockout mice with *Apc*^*Min*^ or *Kras*^*G12D*^ mice to generate intestinal cancer model and lung cancer model, respectively. Tumor progression was monitored, and the influence of *c-mos* deficiency on cancer formation was investigated.

**Specifications table**

**Table:** 

Subject area	Biology
More specific subject area	Tumor biology
Type of data	Figure, Graph
How data was acquired	Real-time PCR, Online Database, Mouse Tumor Model and Histopathological Analysis
Data format	Analyzed
Experimental factors	Histopathological Analysis from both *Apc^*Min*^* intestinal cancer model and *Kras*^*G12D*^ lung cancer model with or without *c-mos* deficiency.
Experimental features	Mouse Tumor Model combined with Online Database Analysis
Data source location	United States
Data accessibility	Data with this article

**Value of the data**

• These data describe the expression of Mos differed between human and mouse.
• These data provide a significant correlation between high Mos expression and poor survival rates in lung cancer patients.
• These data give insights into *c-mos* in tumor progression in both *Apc*^*Min*^ intestinal cancer model and *Kras*^*G12D*^ lung cancer model.
• These data are useful to researchers interested *c-mos* acts as an oncogene.

## 1 Data

The *c-mos* proto-oncogene was one of the first proto-oncogenes to be cloned [1]. Apart from its role in meiosis, many efforts have been made to illuminate the mechanisms by which *c-mos* might acts as an oncogene [2], [3], [4], [5], [6], [7], [8], [9], [10]. *c-mos* or its coding messenger RNA have been confirmed in most somatic tissues at low levels. Increased Mos expression was found in most human tumor tissues. However, a detailed role of *c-mos* in tumor progression remains unknown. This study was aimed to investigate whether *c-mos* is involved in tumor progression, *via* online database analysis and animal tumor models.

We first took advantage of online BIOGPS database (http://www.biogps.org). *c-mos* was found to be expressed almost evenly in human tissues (Fig. 2A), while it expressed significantly higher in ovaries than other tissues in mouse (Fig. 3F). Analysis of the TCGA Lung 2 cohort in the Oncomine database (www.oncomine.org) showed that Mos expression was significantly upregulated in human lung adenocarcinoma samples than in the non-tumorous lung tissues (P<0.01) (Fig. 2B). In addition, analysis of the datasets obtained from Kaplan-Meier survival plotter revealed a significant correlation between high Mos expression and poor survival rates (P<0.01) (Fig. 2C). The expression of Mos in human CRCs implicated that both of human colon and rectal adenocarcinoma tissues had higher Mos expression (P<0.01) (Fig. 2D), although the correlation of high Mos expression with poor survival rates was no trend toward significance (P=0.25) (Fig. 2E).

**Fig. 1:**
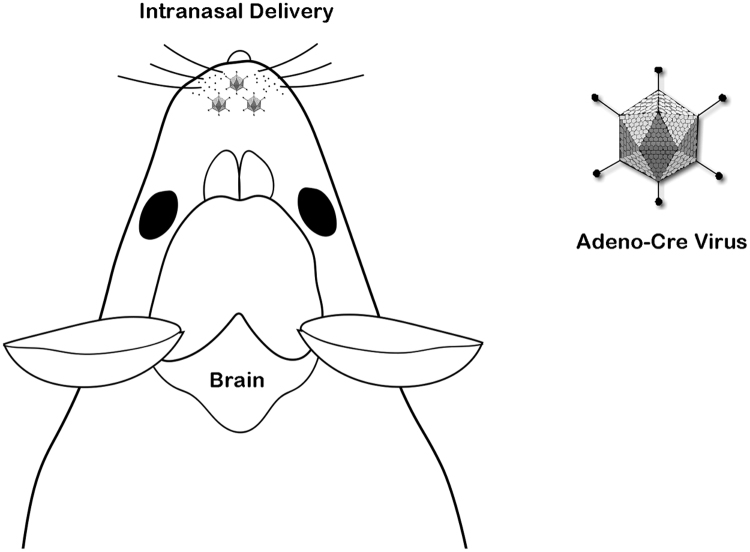
The schematic diagram of intranasal delivery.

**Fig. 2:**
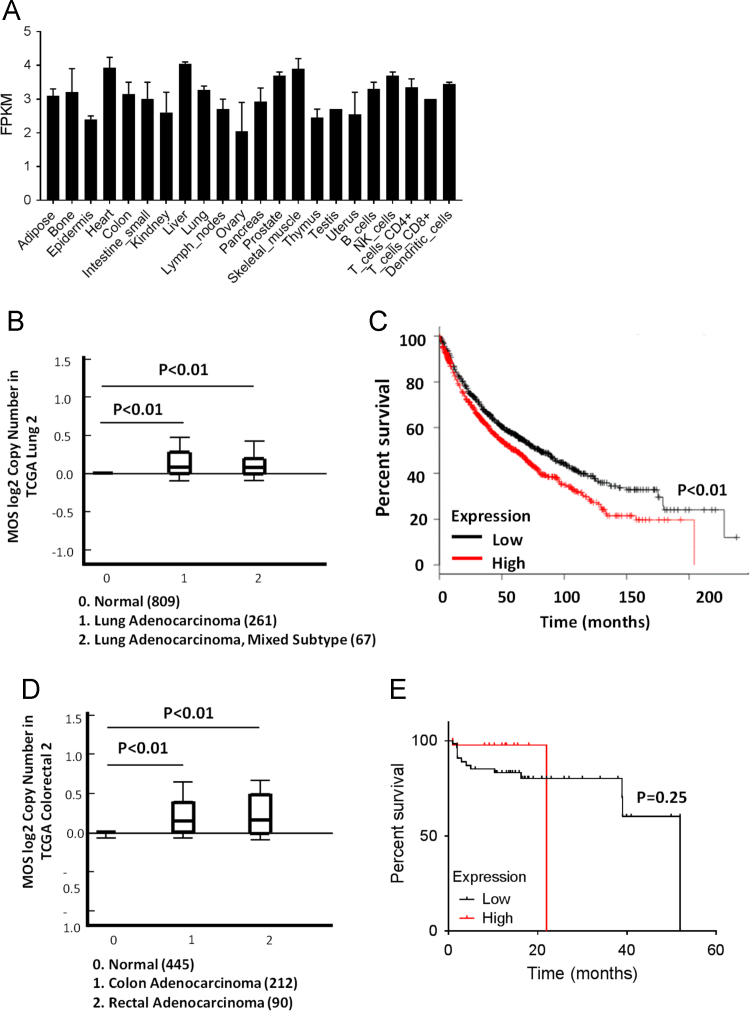
*c-mos* expression in human normal and cancer tissues. (A) Analysis of GeneAtlas U133A, gcrma in the BioGPS database (http://biogps.org) revealed that *c-mos* expression is present in human tissues. (B) Analysis of the TCGA Lung 2 cohort in the Oncomine database (www.oncomine.org) revealed that *c-mos* expression was significantly upregulated in human lung adenocarcinoma samples than the non-tumorous lung tissues. (C) Correlation between *c-mos* expression and patient survival. The *c-mos* expression and overall survival data were obtained from Kaplan-Meier survival plotter datasets as of April 20, 2017. The high and low *c-mos* (221367_at) expressers were grouped using an arbitrary cutoff percentile of 50% (966 for low *c-mos* expressers, and 960 for high c-mos expressers). The Mantel-Cox Log-Rank tests were done using the GraphPad Prism 7 software. (D) Analysis of the TCGA Colorectal 2 cohort in the Oncomine database (www.oncomine.org) revealed that *c-mos* expression was significantly upregulated in human colon and rectal adenocarcinoma samples than the non-tumorous colon tissues. (E) Correlation between *c-mos* expression and patient survival. The *c-mos* expression and overall survival data were obtained from TCGA datasets (Nature 2012). The high and low *c-mos* expressers were grouped using an arbitrary cutoff percentile of 50% (110 for low *c-mos* expressers, and 109 for high *c-mos* expressers). The Mantel-Cox Log-Rank tests were done using the GraphPad Prism 7 software.

**Fig. 3:**
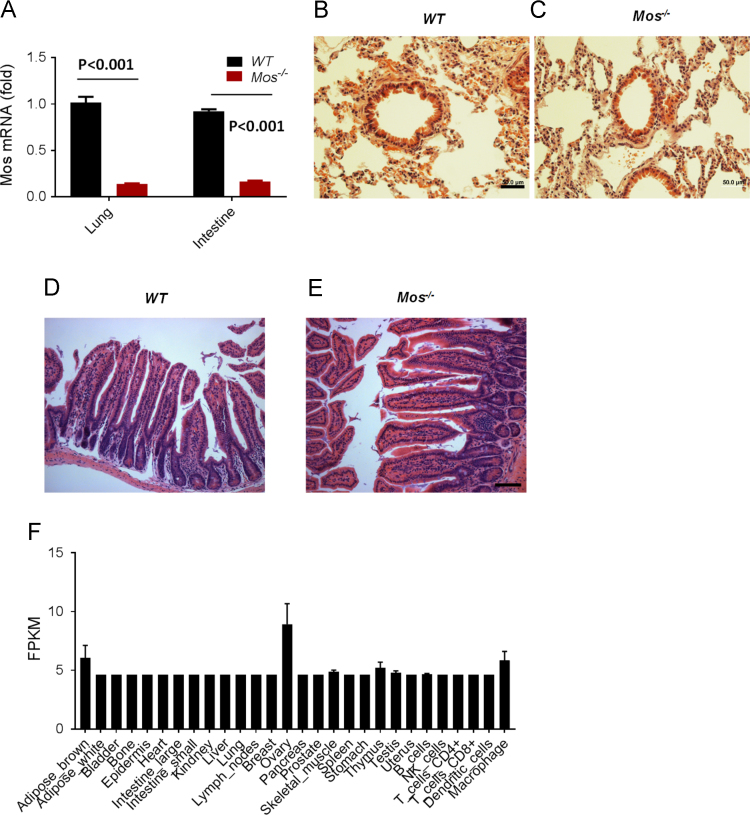
Genetic deletion of *c-mos* gene has no effect on intestine and lung morphogenesis. (A) Real-time PCR quantification of *c-mos* mRNA levels in mouse lung and intestine tissues with wild-type (n=3) and with *c-mos* deficiency (n = 3). Data were presented as means ± SEM. Statistical analyses were performed using Student׳s t-test (B-C) H&E staining of the lung from wild-type and *c-mos*^−/−^ mice with regular architecture. (D-E) Representative H&E staining of intestine from wild-type and *c-mos*^−/−^ mice intestine. Scale bars: 50 μm. (F) Analysis of GeneAtlas MOE430, gcrma in the BioGPS database (http://biogps.org) revealed that *c-mos* expression is present in most mouse tissues but significantly higher in ovaries.

Then, *c-mos* deficiency was confirmed in both of lung and small intestine tissues using Real-time PCR quantification (Fig. 3A). Morphological changes of lung and small intestine in both *Mos*^−/−^ and *WT* mice were observed at the age of 12 months. No significant differences were shown with regard to the tissue size, weight, and macroscopic appearance. The histological structures of lung and small intestine in *Mos*^−/−^ and *WT* mice were nearly identical (Fig. 3B-E).

**Fig. 4:**
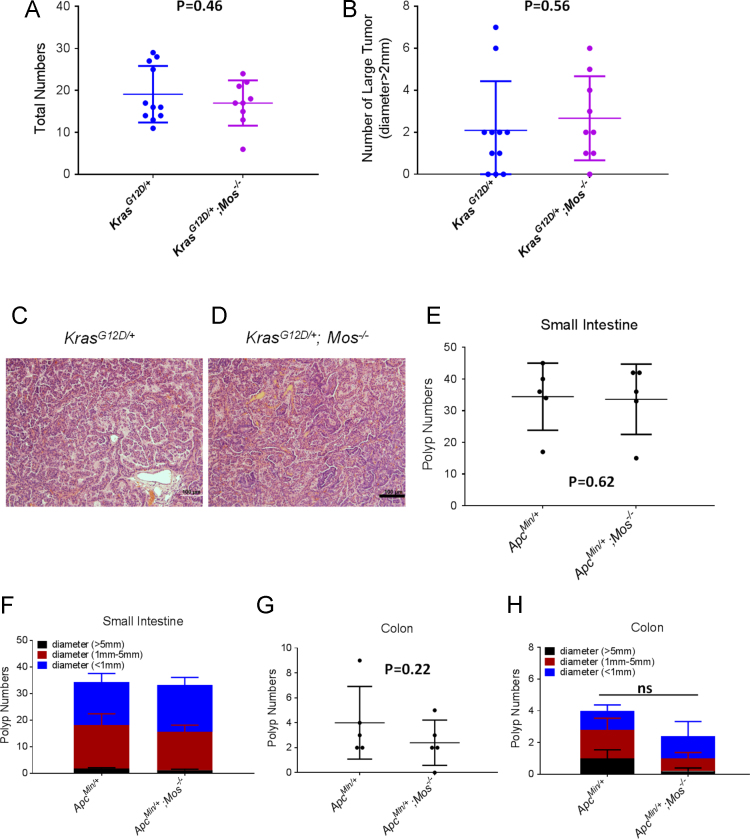
Genetic deletion of *c-mos* gene has on effect on tumor burden in both of *Kras*^*G12D*^ lung cancer mice model and *APC*^*Min/+*^ intestine cancer mice model. (A-B) Tumor development in *c-mos* knockout and wild-type (WT) within *Kras*^*G12D*^ mutation. Animals showed spontaneous lung tumor development at 5 months’ age. A. Total number of lung surface tumor. (B) Number of large tumor based on tumor size (diameter >2 mm). (C-D) Histological confirmation of tumor development. Scale bars: 100 μm. Data are presented as means ± SEM. N=9–11, Student׳s t-test. (E-H). Tumor development in *c-mos* knockout and wild-type (WT) within *APC*^*min/+*^ animals. Animals showed spontaneous intestine tumor development at 5 months’ age. E. Total number of small intestine tumors. F. Number of the intestine tumor based on polys size. G. Total number of colon tumor. H. Number of colon tumor based on polys size. Data are presented as means±SEM. N=5, Student׳s t-test.

To investigate the biological function of Mos in characterized cancers, we cross *Mos*^*−/−*^ with *Kras*^*G12D*^ to generate classic mice lung cancer model. *Kras*^*G12D*^ and *Kras*^*G12D*^*, Mos*^*−/−*^ mice were treated with Adeno-Cre injected intranasally at 8 weeks of age. After 12 weeks, mice were sacrificed for gross inspection and histopathological. Lung tumors were dissected for histopathological analysis. However, there was no difference on the slowdown of tumor progression in *c-mos* deficient mice (Fig. 4A–B). Fig. 4C–D showed representative histological sections from the *Kras*^*G12D*^ mice and the *Kras*^*G12D*^*; Mos*^*−/−*^ mice, which showed no difference on tumor tissue morphogenesis. Then, the role of mos-deficiency in tumor formation was investigated using the *Apc*^*Min/+*^ mouse intestinal tumor model. We found that there was no inhibitory effect on colon tumor progression when the *c-mos* gene was absent (Fig. 4E–F), and the absence of *c-mos* gene had no influence on small intestinal tumor burdens in *Apc*^*Min/+*^ mice neither (Fig. 4G-H).

## 2 Materials and methods

### 2.1 Experimental animals

*Mos*^*tm1Ev*^ (B6.129S6-Mostm1Ev/J), *Apc*^*Min/+*^ (C57BL/6J-ApcMin/J) and *Kras*^*LSL-G12D*^ (B6.129S4-Krastm4Tyj/J) mice were acquired from Jackson Laboratory. *Kras*^*LSL-G12D*^ and *Mos*^*tm1Ev*^ mice were backcrossed to *C57BL/6J* for 3 generations. Lung cancer mice models with *Kras*^*LSL-G12D*^ & *Mos*^*tm1Ev*^ (*Kras*^*G12D*^*, Mos*^*−/−*^) were generated by crossing *Kras*^*LSL-G12D*^ with *Mos*^*tm1Ev*^ mice. Intestine cancer mice model *Apc*^*Min/+*^ & *Mos*^*tm1Ev*^ (*Apc*^*Min/+*^*, Mos*^*−/−*^) mice were generated by crossing *Apc*^*Min/+*^ with *Mos*^*tm1Ev*^ mice. All animals were cared for in strict accordance with National Institutes of Health (USA) guidelines and all procedures were approved by the Yale University Animal Care and Use Committee. IACUC PROTOCOL NUMBER: Mouse Models for Signaling Study (2017–11588).

### 2.2 Mouse tumor model

For *de novo* lung cancer mice model, *Kras*^*G12D*^ (n=11, male), and *Kras*^*G12D*^*, Mos*^*−/−*^ (n=9, male) mice were treated with 2×10^6^ plague-forming unites of Adeno-Cre injected intranasally at 8 weeks of age (weight around 18 g) as previously described [11], [12] (Fig. 1). After 12 weeks, mice were sacrificed in CO2 Rodent Euthanasia Chamber for gross inspection and histopathological. Lung tumors were dissected for histopathological analysis. Intestine cancer mice models with *Apc*^*Min/+*^(n=5, male), *Apc*^*Min/+*^*, Mos*^*−/−*^ (n=5, male) mice were housed for 20 weeks, then mice were sacrificed in CO_2_ Rodent Euthanasia Chamber and intestine tissues were collected for histopathological examination. Tumor number and tumor size were measured. All mice were monitored twice a week until endpoint time of the experiment. No animals were excluded from the analysis.

### 2.3 Quantitative RT-PCR

Lung and intestine samples were collected and total RNA was isolated with RNeasy Plus Mini Kit (QIAGEN) according to the manufacturer׳s instructions. Complementary DNAs were synthesized from the above-mentioned collected RNAs using the iScript cDNA Synthesis Kit (Bio-Rad). Quantitative PCR was done using IQ™ SYBR Green super-mixes and CFX96™ Touch Real-Time PCR detection system (Bio-rad). For all quantitative PCR reactions, Gapdh was measured for an internal control and used to normalize the data. The PCR primers used were as follows: *c-mos*: 5′-CTCCGGAGATCCTGAAAGGA-3′ (sense) and 5′-CAGTGTCTTTCCAGTCAGGG-3′ (antisense). Gapdh: 5′-TGCCCCCATGTTTGTGATG-3′ (forward) and 5′-TGTGGTCATGAGCCCTTCC′ (reverse).

### 2.4 Histopathological analysis

Histopathological analysis was performed according to our previous study [11], [12]. In short, after mice were sacrificed, lungs were inflated with 1 ml Bouin׳s solution (Sigma-Aldrich) at room temperature for 20 min and fixed in 20 ml 4% PFA at 4 °C for 24 h. Fixed lung tissues were embedded in paraffin sectioned at 5 μm thickness for hematoxylin and eosin (HE) staining.

### 2.5 Study design and statistical analysis

Minimal group size for tumor progression studies was calculated using an online power calculator available from DSS Researcher׳s Toolkit (https://www.dssresearch.com/KnowledgeCenter/toolkitcalculators.aspx) with an α of 0.05 and power of 0.8. Animal groups were not blinded but randomized, and investigators were blinded to the tumor counting experiments. No samples or animals were excluded from the analysis. Hypothesis concerning the data which included normal distribution (D׳Agostino-Pearson normality test) and similar variation between the experimental groups were examined for appropriateness before the conduct of statistical tests. All statistical analyses were performed with Student׳s t-test (two independent groups) or two-way ANOVA (multiple groups) using IBM SPSS version 21.0, software (IBM Corp, Armonk, NY).

(1) Analysis of *c-mos* expression in human and mouse tissues from BIOGPS database (http://www.biogps.org): Mouse Dataset: GeneAtlas MOE430, gcrma; Human Dataset: GeneAtlas U133A, gcrma.
(2) Analysis of the *c-mos* expression from Oncomine database (www.oncomine.org)

1) TCGA Lung 2 Dataset Summary

**Table:** 

Title:	The Cancer Genome Atlas - Lung Carcinoma DNA Copy Number Data
Author(s):	TCGA (The Cancer Genome Atlas)
Organization:	The Cancer Genome Atlas, Office of Cancer Genomics, National Cancer Institute, National Institutes of Health, Bethesda, MD 20892.
Reference:	No Associated Paper 2012/10/12
Study Description:	Three hundred sixty-six (366) lung adenocarcinoma samples, 359 squamous cell lung carcinoma samples, 2 paired recurrent lung adenocarcinoma samples, 390 paired normal lung samples, and 420 paired normal blood specimens were analyzed on a custom Agilent microarray. Sample data includes age, TNM stage, sex, survival, smoking status, and others. This dataset is a combination of Lung Adenocarcinoma [LUAD] and Lung Squamous Cell Carcinoma [LUSC] data from the TCGA data portal and consists of Level 3 data (segmented using CBS). The resulting segments were mapped to RefSeq gene coordinates as provided by UCSC (UCSC refGene, July 2009; hg18, NCBI 36.1, March 2006). The samples were originally run on the Affymetrix SNP 6.0 platform. Corresponding gene expression data is available in TCGA Lung.
Array Type(s):	RefSeq Genes (UCSC refGene, July 2009, hg18, NCBI 36.1, March 2006) Measured 18,823 genes, 19,084 reporters.
Experiment Type:	DNA
Data Link(s):	http://tcga-data.nci.nih.gov/tcga/
2) TCGA Colorectal 2 Dataset Summary

**Table:** 

Title:	The Cancer Genome Atlas - Colon and Rectum Adenocarcinoma DNA Copy Number Data
Author(s):	TCGA (The Cancer Genome Atlas)
Organization:	The Cancer Genome Atlas, Office of Cancer Genomics, National Cancer Institute, National Institutes of Health, Bethesda, MD 20892.
Reference:	No Associated Paper 2011/09/09
Study Description:	Four hundred thirty-six (436) colorectal adenocarcinoma and 351 paired normal blood and 94 paired normal colorectal tissue samples were analyzed. Sample data includes age, histology, microsatellite status, TNM stage, KRAS and BRAF mutation status, sex, stage, and others. This dataset is a combination of Colon Adenocarcinoma [COAD] and Rectum Adenocarcinoma [READ] data from the TCGA data portal and consists of Level 3 data (segmented using CBS). The resulting segments were mapped to RefSeq gene coordinates as provided by UCSC (UCSC refGene, July 2009; hg18, NCBI 36.1, March 2006). The samples were originally run on the Affymetrix SNP 6.0 platform. Corresponding gene expression data is available in TCGA Colorectal.
Array Type(s):	RefSeq Genes (UCSC refGene, July 2009, hg18, NCBI 36.1, March 2006)
	Measured 18,823 genes, 19,084 reporters.
Experiment Type:	DNA
Data Link(s):	http://tcga-data.nci.nih.gov/tcga/

(3) Correlation of Mos expression and patient survival in lung cancers
  The Mos expression and overall survival data were obtained from Kaplan-Meier survival plotter datasets as of April 20, 2017. (http://kmplot.com/analysis/index.php?p=service&cancer=lung). The high and low Mos (221367_at) expressers were grouped using an arbitrary cutoff percentile of 50% (966 for low Mos expressers, and 960 for high Mos expressers). Pearson׳s correlation coefficient for Mos expression and patients’ mortality is -0.78, p-value is 0.0024. The Mantel-Cox Log-Rank tests and Correlation tests were done using the GraphPad Prism 7 software.
(4) Correlation of Mos expression and patient survival in colorectal cancers

The Mos expression and overall survival data were obtained from TCGA datasets (The Cancer Genome Atlas Network, 2012). The high and low Mos expressers were grouped using an arbitrary cutoff percentile of 50% (110 for low Mos expressers, and 109 for high Mos expressers). Pearson׳s correlation coefficient for Mos expression and patients’ mortality is −0.34, p-value is 0.25. The Mantel-Cox Log-Rank tests and Correlation tests were done by using GraphPad Prism 7 (GraphPad Software, La Jolla, CA, USA).
