# Supplementary material for: Data on tumor progression of c-mos deficiency in murine models of KrasG12D lung and ApcMin colorectal cancer
Source: Data Brief. 2018 Aug 31;20:1124–32. doi: 10.1016/j.dib.2018.08.129 (PMC6139997; doi:10.1016/j.dib.2018.08.129)
Supplement: Supplementary file 1 — Supplementary material. [file mmc1.pdf]

CONFLICT OF INTEREST FORM

We wish to confirm that there are no known conflicts of interest associated with this publication and there has been no significant financial support for this work that could have influenced its outcome.

We confirm that the manuscript has been read and approved by all named authors and that there are no other persons who satisfied the criteria for authorship but are not listed. We further confirm that the order of authors listed in the manuscript has been approved by all of us.

We confirm that we have given due consideration to the protection of intellectual property associated with this work and that there are no impediments to publication, including the timing of publication, with respect to intellectual property. In so doing we confirm that we have followed the regulations of our institutions concerning intellectual property.

We further confirm that any aspect of the work covered in this manuscript that has involved either experimental animals or human patients has been conducted with the ethical approval of all relevant bodies and that such approvals are acknowledged within the manuscript.

We understand that the Corresponding Author is the sole contact for the Editorial process (including Editorial Manager and direct communications with the office). She is responsible for communicating with the other authors about progress, submissions of revisions and final approval of proofs.

Signed by the corresponding author
Qian Xiao

Qian Xiao

April 5th, 2018
